# Causes of Ring-Related Leg Injuries in Birds – Evidence and Recommendations from Four Field Studies

**DOI:** 10.1371/journal.pone.0051891

**Published:** 2012-12-26

**Authors:** Michael Griesser, Nicole A. Schneider, Mary-Anne Collis, Anthony Overs, Michael Guppy, Sarah Guppy, Naoko Takeuchi, Pete Collins, Anne Peters, Michelle L. Hall

**Affiliations:** 1 Institute of Ecology and Evolution, Department of Biology, University of Bern, Bern, Switzerland; 2 Department of Ecology, Swedish University of Agricultural Sciences, Uppsala, Sweden; 3 Anthropological Institute and Museum, University of Zurich, Zurich, Switzerland; 4 School of Zoology, University of Tasmania, Hobart, Tasmania, Australia; 5 Iver, Buckinghamshire, United Kingdom; 6 Hawker, Australian Capital Territory, Australia; 7 Moruya, New South Wales, Australia; 8 Fujiwasa, Kanagawa, Japan; 9 Soldiers Hill, Ballarat, Victoria, Australia; 10 School of Biological Sciences, Monash University, Clayton, Victoria, Australia; 11 Department of Zoology, University of Melbourne, Melbourne, Victoria, Australia; Institut Pluridisciplinaire Hubert Curien, France

## Abstract

One of the main techniques for recognizing individuals in avian field research is marking birds with plastic and metal leg rings. However, in some species individuals may react negatively to rings, causing leg injuries and, in extreme cases, the loss of a foot or limb. Here, we report problems that arise from ringing and illustrate solutions based on field data from Brown Thornbills (*Acanthiza pusilla*) (2 populations), Siberian Jays (*Perisoreus infaustus*) and Purple-crowned Fairy-wrens (*Malurus coronatus*). We encountered three problems caused by plastic rings: inflammations triggered by material accumulating under the ring (Purple-crowned Fairy-wrens), contact inflammations as a consequence of plastic rings touching the foot or tibio-tarsal joint (Brown Thornbills), and toes or the foot getting trapped in partly unwrapped flat-band colour rings (Siberian Jays). Metal rings caused two problems: the edges of aluminium rings bent inwards if mounted on top of each other (Brown Thornbills), and too small a ring size led to inflammation (Purple-crowned Fairy-wrens). We overcame these problems by changing the ringing technique (using different ring types or larger rings), or using different adhesive. Additionally, we developed and tested a novel, simple technique of gluing plastic rings onto metal rings in Brown Thornbills. A review of studies reporting ring injuries (N = 23) showed that small birds (<55 g body weight) are more prone to leg infections while larger birds (>35 g) tend to get rings stuck over their feet. We give methodological advice on how these problems can be avoided, and suggest a ringing hazard index to compare the impact of ringing in terms of injury on different bird species. Finally, to facilitate improvements in ringing techniques, we encourage online deposition of information regarding ringing injuries of birds at a website hosted by the European Union for Bird Ringing (EURING).

## Introduction

Leg ringing is an essential technique in field ornithology and is currently used on a large number of bird species worldwide. This methodology has advanced our understanding of the ecology, behaviour, life-histories and evolution of birds [1], [2], [3], [4]. Numbered metal rings are durable, allowing individual birds to be uniquely identified over the course of their life, and research effort and records to be coordinated at a national level. Colour rings allow individuals to be distinguished in the field without recapture, an approach started in the nineteen thirties [5] that has become widespread since. A large number of detailed studies on bird species have relied on this technique to distinguish individuals in the field [2], [6].

Given that leg ringing is widespread, it is important to identify potential negative effects of this method on the birds under study [7]. Negative impacts not only pose an ethical problem, but may also skew research results, especially if particular sub-groups of birds are more affected by ringing than others (for example only breeding females, see results below). In this paper we focus only on leg rings and not alternative marking methods such as wing-tagging, nostril rings or neck collars (see [7] for a discussion of effects of alternative marking methods).

The negative effects of colour-ringing are mainly influenced by the size and material of the rings [8], [9], [10], as well as the environment and behaviour of the species under study [7], [11]. Appropriate ring sizes have already been determined for many species [7], but are often unknown in parts of the world where little bird research has been conducted. Currently, the most commonly used material to produce colour rings is plastic, mainly PVC (sold under different trade names, e.g. Darvic or Salbex), but also PMMA (polymethylmethacrylate), while celluloid rings are no longer manufactured. PVC rings are durable and can last for more than a decade on birds (at least in temperate environments with low UV levels; Michael Griesser, personal observations), but they are known to have negative effects on the health of certain species (see Table 1). PMMA rings have so far been used mainly on larger species but are also a suitable alternative for smaller species [12], although their impact on the health of small birds remains unstudied.

**Table 1:** Overview of species (listed by increasing body weight) reported to have problems caused by plastic or metal rings.

species	taxonomic group	mass (g)	years studied	foot/leg infections	ring stuck over foot	toes trapped in rings	ringing hazard a	solution	reference
Brown Thornbill (NSW) *Acanthiza pusilla*	Pardalotidae	6.7	7	potentially 100% b	-	-	potentially high b	avoid using metal over metal rings	this study
Brown Thornbill (TAS) *Acanthiza pusilla*	Pardalotidae	7.1	5	3%	-	-	0.30	avoid using plastic rings	this study, [19]
Buff-rumped Thornbill *Acanthiza reguloides*	Pardalotidae	7.1	n.a.	yes	-	-	n.a.	avoid using plastic rings	[19]
Yellow-rumped Thornbill *Acanthiza chrysorrhoa*	Pardalotidae	8.25	n.a.	yes	-	-	n.a.	avoid using plastic rings	[19]
Purple-crowned Fairy-wren *Malurus coronatus*	Maluridae	10.5	5	1.4%	-	-	0.11	increase ring size, avoid two plastic rings on one leg	this study
Golden-cheeked Warbler *Dendroica chrysoparia*	Passeroidea	10.8	n.a.	7.5%	-	-	0.07	none	[42]
Black-naped Monarch Hypothymis azurea	Monarchidae	11.2	2	35.3%	-	-	0.19 c	avoid using plastic rings	[10]
Madagascar Paradise-flycatcher Terpsiphone mutata	Monarchidae	12.2	3	13.4%	-	-	0.19 c	avoid using plastic rings	[10]
Ochre-bellied Flycatcher *Mionectes oleaginous*	Tyrannida	12.5	n.a.	yes	-	-	n.a.	avoid using plastic rings	[13]
Willow Flycatchers *Empidonax trailIii*	Tyrannida	13.8	7	9.6%	-	-	1.06	avoid using plastic rings	[43]
Pied Flycatcher Ficedula hypoleuca	Muscicapidae	15.9	2	9.5%	-	-	0.19 c	none	[10]
Black-headed Paradise Flycatcher Terpsiphone rufiventer	Monarchidae	16.5	5	16.7%	-	-	0.19 b	none	[10]
Spotted Flycatcher Muscicapa striata	Muscicapidae	16.6	5	16.7%	-	-	0.19 c	avoid using plastic rings	[10]
Blue-shouldered Robin Chat *Cossypha cyanocampter*	Muscicapoidea	30	n.a.	yes	-	-	n.a.	n.a.	[13]
Semi palmated Sandpiper *Calidris pusilla*	Charadrii	30.5	8	0.4%	-	-	0.04	use larger ring	[8]
Bell Minor *Manorina melanophrys*	Meliphagidae	32	<10	8%	-	-	0.22	use larger rings	[9]
Hihi *Notiomystis cincta*	Meliphagidae	32	<10	54% d	-	-	5.4	avoid using plastic rings	[30]
North Island Robin *Petrocia longipes*	Petroicidae	35	4	-	-	2%	0.11	colour rings removed	[37]
Spotted Sandpiper *Actitis macularia*	Charadrii	41.5	19	-	3% e	-	0.27	none	[11]
Kentish Plover *Charadrius alexandrinus*	Charadrii	42		1.9%	-	-	1.1	avoid attaching ring to the tarsus	[32]
Piping Plover *Charadrius melodus*	Charadrii	55		10.7%	-	-	1.0	avoid using tall anodized rings	[31]
Siberian Jay *Perisoreus infaustus*	Corvoidea	85	<20	-	0.2%	-	0.04	use plastic rings with 2.5 wraps, change glue	this study
Common Tern *Sterna hirundo*	Charadrii	116	10	-	1% f	-	0.26	none	[39]

**a**: The ringing hazard is calculated as the total of hazard points for all individuals in the study population (see Table 3) divided by the total number of individuals.

**b**: Brown Thornbills in New South Wales (N = 20 individuals, see methods above) were ringed with 3 anodized aluminium rings and a numbered magnesium alloy ring. The edges of the aluminium rings bent inwards, but given that these rings were removed in time, none of the birds suffered from damages. However, it is likely to assume that the inward bent edges would have damaged the legs eventually if not removed.

**c**: No detailed data available for the different species.

**d**: 54% of 40 individuals translocated to new location.

**e**: Toe loss due to plastic ring stuck over foot (0.4%), foot loss due to metal ring (2.6% of all individuals).

**f**: One individual had a plastic ring stuck over the tibo-tarsal joint.

Since plastic rings may have a negative impact in some bird species, it is necessary to closely monitor ringed individuals and seek alternative marking methods for those species that do not tolerate plastic colour rings. A few alternative solutions to plastic colour rings are available. The only commercially available option is plain anodised aluminium rings (such as those produced by AC Hughes, Hampton Hill, Middlesex, UK). However, these rings are soft anodised and the colour typically wears off within a couple of years (Michael Griesser, personal observations; David Green, personal communication). Hard anodised coloured metal rings would be more durable, but are not commercially available due to higher production costs. Further limitations of coloured aluminium rings are highlighted in the results section. As an alternative option, metal rings can be coloured by the application of coloured auto pin-striping tape and flexible epoxy [13]. This method is very laborious and hence not commercially available, but provides a useful alternative as discussed below.

Based on this background, we first report several problems observed in four long-term field studies of three species which differ in their ecology and live in contrasting environments: Brown Thornbills *Acanthiza pusilla*; Siberian Jays *Perisoreus infaustus*; and Purple-crowned Fairy-wrens *Malurus coronatus*. We then describe a simple alternative field method to colour ring birds that are sensitive to plastic rings. Finally, we suggest methodological changes that can be made to reduce injuries, and provide suggestions to minimise the impact of colour rings on the health status of birds.

## Methods

### Study systems

#### 1. Brown Thornbills

Brown Thornbills were studied from 2009 to 2011 by Michael Griesser, Nicole A Schneider, Mary-Anne Collis and Naoko Takeuchi to investigate the link between parental behaviour and nest predation [14], [15] (ethics licence A00110979 University of Tasmania Animal Ethics Committee). The study site next to Launceston, Tasmania, Australia (41°26′S, 147°05′E) has a temperate climate with a mean annual rainfall of 784 mm, a mean minimum temperature of +2°C during winter, and a mean maximum temperature of +24°C during summer. The vegetation consists of native woodland with mainly eucalypt (*Eucalyptus* spp.) and wattle (*Acacia* spp.) stands and an understory of large tussock grasses and bracken ferns. Fieldwork took place each year during the breeding season (October-December) and during the post-breeding season (January). During the field season birds were observed at least once a week.

In addition, Brown Thornbills were studied from 2006 onwards by Anthony Overs, Michael Guppy and Sarah Guppy in a long-term ecological study of woodland birds in Australian coastal forests [16] (Australian Bird and Bat Banding Scheme (ABBBS) licence number 2995 and 2857). The study site at Maulbrooks Road, Moruya, New South Wales, Australia (35°52′S, 150°03′E) also has a temperate climate with a mean annual rainfall of 952 mm, a mean minimum temperature of +6°C during winter, and a mean maximum temperature of +24°C during summer. The vegetation in the study area consists of native woodland dominated by eucalypts (*Eucalyptus* spp.) and wattles (*Acacia* spp.) while the understory consists of myrtle and casuarina thickets [17]. Birds were caught twice per year, once in October and once in December. Observations (about 3 hrs each) took place continuously throughout the breeding season (from August 1^st^ to January 31^st^) on most days (about 90% of days).

**Figure 1:**
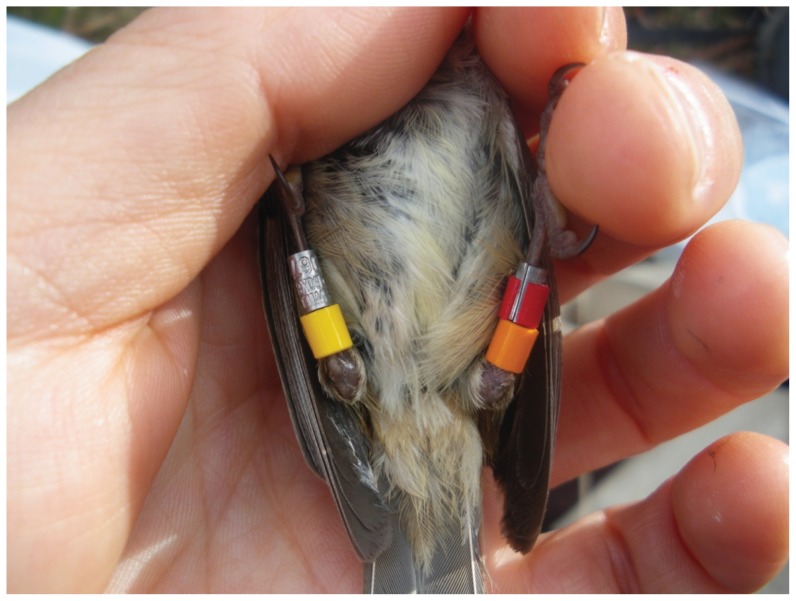
Brown Thornbill where the leg on the right of the picture is ringed with a metal-on-plastic ring, with the metal protruding so the plastic does not make contact with the foot. Thornbills are known to react negatively to plastic colour rings that touch the feet. While the metal-on-plastic ring method solved this problem, Brown Thornbills on Tasmania reacted to plastic colour rings in upper position (see Fig. 4).

Since prior studies on Brown Thornbills in Canberra [18] have shown that plastic rings may cause inflammations when in contact with the foot [19], we were given permission from the ABBBS to test a novel ringing technique (plastic-on-metal ring) at our site in Tasmania, described in detail in section 4 of the Results. Birds were ringed with one of these plastic-on-metal rings (see Fig. 1) on one leg, and a numbered aluminium magnesium alloy ring (size 01; 2 mm inner diameter) on the other leg. A single plastic split ring (PVC or celluloid) was fitted above each of these (i.e. plastic ring not adjacent to the foot; Fig. 1; Table 2). Given that Brown Thornbills in Tasmania are 10–20% larger than on the mainland [20], we used plastic split rings with an inner diameter of 2.7 mm instead of the smallest plastic ring size from AC Hughes with an inner diameter of 2.3 mm to avoid problems of materials accumulating under the rings (see below).

**Table 2:** Body measures and ring measures of the study species.

	Siberian jay	TAS Brown Thornbill	NSW Brown Thornbill	Purple-crowned Fairy-wren
weight (g) mean±SE (min-max)	85.7±0.3 (74.5–102)	8.0±0.09 (6.2–9.5)	6.7±0.14 (6.0–7.5)	10.6±0.03 (8.3–13.8)
tarsus diameter mid (mm)	3.6±0.03	n.a.	n.a.	1.88±0.01
tarsus diameter foot (mm)	3.8±0.03	n.a.	n.a.	2.14±0.02
metal ring inner diameter (mm)	5.0	2.0 (size 01)	2.0 (size 01)	2.0 (size 01) 2.3 (size 02)
colour ring inner diameter (mm)	5.5	2.7 (size XCS)	2.3 (size XF)	2.3 (size XF)
weight metal ring (g)	0.7 (size 5)	0.04 (size 1)	0.04 (size 1)	0.04 (size 1)
weight plastic ring (g)	0.18	0.025	0.02	0.02
weight split-colour metal ring (g)	-	-	-	0.07
% total ring weight in relation to mean body weight	1.4%	1.4% a	2.3% c	1.0% e
		1.9% b	1.5% d	1.1% f

**a**: 1 alloy ring and 3 plastic colour rings.

**b**: 1 alloy ring, 1 plastic-on-metal ring and 2 plastic colour rings.

**c**: 1 alloy ring and 3 coloured aluminium rings.

**d**: 1 alloy ring and 3 plastic colour rings.

**e**: 1 alloy ring and 3 plastic rings.

**f**: 1 anodised alloy ring and 1 split-colour ring.

At the coastal New South Wales site, Brown Thornbills were ringed with a numbered magnesium alloy ring (size 01, 2 mm inner diameter) and three plastic (PVC or celluloid) split rings (inner diameter of 2.3 mm from AC Hughes), one on top of the alloy ring and two on the other leg (Table 2). In addition we were authorized by the ABBBS to ring 20 individuals with metal rings only (2 on each leg). Here we used soft anodized aluminium rings size 1/2 with an inner diameter of 2.0 mm from AC Hughes.

#### 2. Siberian Jays

Siberian Jays were studied from 1989 onwards by Jan Ekman (Uppsala University, Sweden), Michael Griesser and colleagues to investigate the evolution of family living and the interplay between habitat structure, nest predation and population dynamics [21], [22], [23] (under the licence from Umeå djurförsöksetiska nämnd license numbers A80-99 and A504). The study site near Arvidsjaur, Sweden (65°40′N, 19°0′E) [21] is about 100 km south of the Arctic Circle, and has a mean annual rainfall of 650 mm, a mean minimum temperature of −18°C during winter (with temperatures down to −50°C), and a mean maximum temperature of +18°C during summer. The vegetation at the study site consists of a mosaic of managed and unmanaged northern boreal forest [24]. Fieldwork took place during most months, with a focus on the reproductive season (March–May) and autumn (September–October).

**Figure 2:**
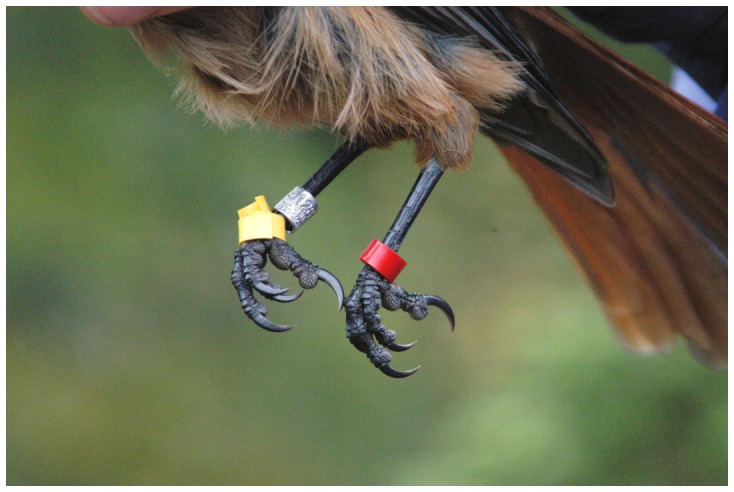
Siberian jay with partly unwrapped wrap-around colour ring stuck over the foot. The yellow plastic ring was mounted on top of the metal ring but slipped over the metal ring when the bird tried to remove the plastic ring. This individual was colour ringed as a juvenile in autumn 2002, and recaptured in October 2003 with the colour ring partly stuck over the foot. It subsequently dispersed in spring 2004 to the neighbouring group where it became a breeder.

All birds were ringed with a numbered stainless steel ring (Swedish size 5; Table 2) and with 2–3 plastic rings, one above the metal ring and one or two on the other leg. We used both celluloid and PVC wrap-around colour rings (flatband style, referred to as wrap-around ring hereafter; illustrated in Fig. 2). In total, we colour ringed 1258 individuals between 1989 and 2011. Between 1989 and 2004 we used PVC wrap-around rings produced by PJ Andersson (Bankerud, Sweden; inner diameter 5.5 mm, corresponding to size 5 Swedish rings; 2.5 wraps) and from 2004 onwards, we used both celluloid and PVC wrap-around rings produced by AC Hughes (size FB, inner diameter 5.5 mm, 1.8–2.0 wraps). Birds were observed regularly and recaptured at least once every year, when their legs were inspected, and any problems or injuries associated with the colour rings recorded (i.e. colour rings missing, colour rings stuck over the toes). We measured tarsus widths of 38 birds at the midpoint of the tarsus and distally at the last undivided scale before the toes diverge (as illustrated at Fig. 1 in [9]).

#### 3. Purple-crowned Fairy-wrens

Purple-crowned Fairy-wrens were studied from 2005 to 2010 (with annual censuses in two years thereafter), by Sjouke A Kingma, Anne Peters and Michelle L Hall for studies on cooperative breeding and acoustic communication [25], [26] (Max Planck Institute for Ornithology Animal Ethics Committee, and Western Australia Department of Conservation and Environment licences SF007544 and BB002798). The study site at Mornington Wildlife Sanctuary in the Kimberley, Western Australia, Australia (17.5°S, 126.1°E), has a tropical climate with most of the 752 mm annual rainfall falling in a four-month wet season (December–March). The mean minimum temperature in July is +11°C and the mean maximum temperature in November is +41°C. Purple-crowned Fairy-wrens are riparian specialists, and at our site occupied sections of the creeks that were lined with river pandanus (*Pandanus aquaticus*). Birds were re-sighted weekly throughout the year.

Birds were ringed with a numbered ABBBS magnesium alloy ring, initially using size 01 rings (internal diameter = 2.0 mm) recommended by the ABBBS, and later size 02 rings (internal diameter = 2.3 mm) (Table 2). For individual identification in the field, we first used a unique combination of three coloured plastic split rings (PVC or celluloid, internal diameter = 2.3 mm), one above the metal and two on the other leg, but subsequently replaced the plastic ring near the foot with a metal ring. From December 2008, all newly captured birds were ringed with a single metal ring on each leg (i.e. no plastic rings used): an anodised numbered ABBBS metal ring and a split-colour metal ring (made according to [13]). We measured tarsus widths of all birds using the same method as described above for the Siberian jays. We checked for signs of injuries in birds in the study area by weekly population census, and checked the legs of all captured birds for injuries or accumulation of material on the legs under the rings.

## Results

### 1. Injuries caused by plastic rings

We observed three different problems caused by plastic rings: (a) inflammations due to material accumulating under the rings, (b) contact inflammations, and (c) toes or the foot getting trapped in partly unwrapped wrap-around rings.

#### (a) Leg inflammations caused by material accumulating under rings

**Figure 3:**
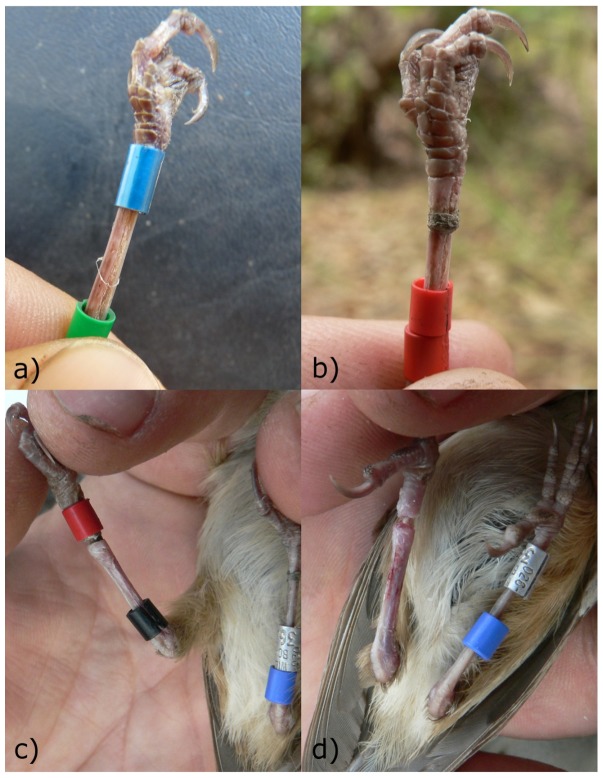
Likely sequence leading to leg injuries in Purple-crowned Fairy-wrens. A fine bracelet of spider web caught on leg (a) attracted other material to form a constricting ring around the leg (b) which gradually cut into the leg causing swelling (c). Injuries usually healed rapidly if rings and constricting material were removed early enough (d). The constriction could form above, below, or underneath a ring, but often caused worse swelling if it was under the ring and close to the foot.

Purple-crowned Fairy-wrens suffered from leg inflammations due to material accumulating under plastic rings. We ringed 269 adults and 522 nestlings from July 2005 to December 2010. Over a six month period from June 2008, we observed leg injuries in 11 birds (5.6% of 191 birds present in the population at the time) that had been ringed for an average of 2.3 years (range = 5.9 months to 3.3 years) and these injuries were on the leg with two coloured plastic rings in 91% of cases (10 of 11). Eight cases were females (six breeders, representing 10.2% of female breeders at that time) and three were males (all breeders, representing 5.2% of male breeders at that time). We removed all rings from the injured leg, and also removed a ring of material that had accumulated on the leg (sometimes cutting into the leg to the bone; Fig. 3d). The foot was already dead by the time we were able to catch the bird in one of the 11 cases (9%). Survival following ring removal was bimodal: five birds survived 0–6 months (mean 2.5 months; 7 weeks for the bird whose foot had already died) and six birds survived 1.2–3.6 years (mean 2.1 years).

Following close inspection of the legs of many recaptured and newly-captured Purple-crowned Fairy-wrens, we suggest the following sequence led to injuries (illustrated in Fig. 3). A ‘bracelet’ of spider web sometimes formed around a bird's leg (also observed on an un-ringed bird) which, if not preened off, gradually tightened on the leg and attracted other material. We observed and removed such a ring of material on the leg (ranging from a tiny thread of spider web to a large accumulation) from 33 birds that had no leg injury. The ring of material was usually on the leg with two plastic rings (18 cases), on the leg with plastic above metal (6 cases), or on both legs (5 cases) and unknown in 4 cases. Constriction of the leg by the ring of material occurred before accumulated debris impeded free movement of a ring, but swelling and injury worsened once ring movement ceased, and could lead to loss of a foot if both the ring and the material constricting the leg were not removed early enough (1 case, described above). The injuries may have been more common on legs with two plastic rings because plastic rings generate more static than metal rings [9] and may therefore be more likely to attract and accumulate other material under the ring. Females use spider webs as nesting material in the early stages of nest-building, which may explain why more females suffered injuries.

#### (b) Contact inflammations

**Figure 4:**
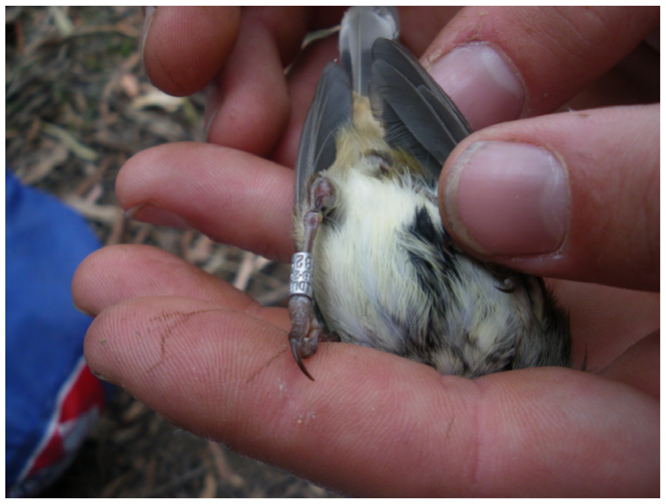
Injuries on Brown Thornbill legs caused by plastic colour ring in upper position. Since Brown Thornbills in Tasmania often forage hanging upside down, the plastic ring is pushed onto the tibo-tarsal joint, causing a severe infection in 3% of birds, and loss of a foot in 1%. This individual was initially colour ringed on 13 December 2010, and recaptured to remove the colour rings on 9 April 2011.

As known from earlier studies [19], Brown Thornbills are among the species known to suffer from inflammations when plastic rings are in contact with their feet. Thus, in the Tasmanian population we placed the novel plastic-on-metal ring (method described below) in the basal position on the leg which did not carry the numbered alloy ring. We colour ringed 117 individuals in December 2009, and 197 individuals between October and December 2010 using plastic-on-metal rings and plastic colour rings. While the few Brown Thornbills we recaptured in October 2010 did not show any sign of reaction towards the plastic rings, recapture of birds between November and December 2010 revealed a new problem which Brown Thornbills developed as a consequence of plastic colour rings in the upper position. Instead of causing infections at the foot, the birds developed infections at and above the tibio-tarsal articulation on both legs. This is likely to be a result of Brown Thornbills in Tasmania using a different foraging technique than birds on the mainland. Tasmanian Brown Thornbills spend a substantial amount of time hanging upside down to glean insects off branches or leaves [27], whereas birds on the mainland more often forage perched on leaves or branches [28]. As a consequence of this foraging behaviour the plastic colour ring in the upper position is in regular contact with the tibio-tarsal joint, causing inflammations (Fig. 4). Also, the larger weight of the plastic-on-metal ring (Table 2) is likely to increase the force which pushes the plastic ring onto the tibo-tarsal joint. In one case a plastic ring indeed slipped over the tibo-tarsal joint and was stuck on the tibia, which highlights the importance of choosing an appropriate ring size (not too large or small).

In mid-December 2010, immediately after this problem first became apparent, we began recapturing birds, and successfully recaptured and removed colour rings on a total of 78 birds in December 2010, January 2011, and April 2011. Some of the birds which had swollen legs had accumulated material around the leg, forming a ‘bracelet’ as observed in the Purple-crowned Fairy-wrens (see above). However, we were unable to identify this material in the field. Given that most birds were ringed as juveniles, a large proportion was likely to have died due to natural reasons, or dispersed off the study site [18], [29]. After the capture session of April 2011, we observed an additional 20 colour-ringed individuals which we were unable to catch, of which two had swollen legs. In total, of the 314 birds ringed between December 2009 and December 2010, 1% of birds lost one limb, and 2% had swollen legs.

In contrast to Tasmanian Brown Thornbills and those in a study population in Canberra (David Green, personal communication) [19], we did not observe any leg or foot injuries in the coastal New South Wales population on the 113 individuals wearing three plastic rings (one in the basal position touching the foot) from September 2008 onwards. Two individuals which were ringed with 3 plastic rings in September 2008 were still present in August 2012 and did not show any injuries. Moreover, given the frequent re-observations of the ringed individuals between August and January (mean±SE = 21.2±2.6 re-observations per season), and a within-season re-capture rate around 60%, it is unlikely that we overlooked birds that developed injuries from plastic rings.

#### (c) Toes or foot getting trapped in partly unwrapped wrap-around rings

While smaller bird species are usually ringed with plastic split rings, which normally fall off when a bird manages to remove a ring, larger birds are typically ringed with wrap-around plastic colour rings (flatband style). Although such rings are not as easily removed by birds as split rings, they may get stuck over the foot or the leg if the bird succeeds in partially unwrapping it (Fig. 2).

We used plastic wrap-around (PVC and celluloid) colour rings on Siberian Jays. Out of the 285 ringed Siberian Jays present during summer 2011, 17 individuals (6%) had removed 1–2 colour rings (N = 20 rings in total), even though the rings were glued with cyanoacrylate superglue when birds were ringed. Individuals that managed to remove rings were more likely to have been ringed as adults than as nestlings (N birds removing rings: ringed as adults = 16 out of 169, N ringed as nestlings = 1 out of 116; Fisher Exact test: p = 0.0035). However, it appears that ring removal was not dependent on the time passed since ringing.

There was no difference in the frequency with which birds removed PVC or celluloid rings (PVC rings: 16 out of 563 rings removed, celluloid rings: four out of 263 rings removed; Fisher Exact test: p = 0.33). While PVC rings are more rigid than celluloid rings and thus less easy to remove than celluloid rings, cyanoacrylate superglue often seals PVC rings for less than a year whereas celluloid rings are glued together more permanently.

If individuals only manage to partly unwrap wrap-around plastic rings, those may get stuck over their foot. We observed this problem in nine out of 811 Siberian Jays (1.1%) between 1998 and 2011. Most of these incidents occurred after we started to use colour rings with 1.8–2.0 wraps instead of 2.5 wraps (8 out of 333 versus 1 out of 481; Fisher Exact test: p = 0.005). Two Siberian jays (0.2%) were injured as a consequence of carrying plastic rings. In one case a bird lost a toe after that got stuck under the ring (spring 2011) before losing its entire foot after the ring had been removed, possibly from a secondary infection. This bird disappeared during autumn 2012. Another bird which got the ring trapped over the foot had a minor infection under the ring, and no longer used the foot even one year after the ring was removed. The four other birds did not show any sign of injuries after the ring had been removed.

In addition, about one third of all ringed Siberian Jays showed minor abrasion on their feet. However, we also observed the same type of abrasions on un-ringed birds, suggesting that they were not caused by the rings. These abrasions never led to an infection.

### 2. Injuries caused by metal rings

We encountered two problems as a consequence of birds carrying metal rings: (a) the edges of the rings bend inwards or (b) the rings were too small and hence caused inflammations.

#### (a) Inward-bending edges of aluminium rings

**Figure 5:**
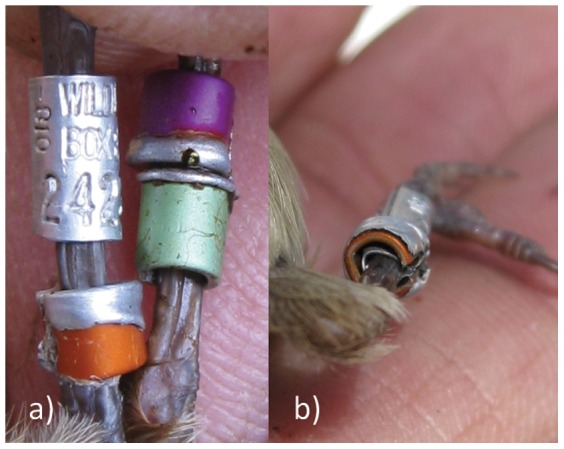
Brown Thornbills with damaged aluminium rings which have been mounted on top of each other. Individual a) was ringed in September 2007 and re-captured the beginning of October 2008 to remove the aluminium rings and mount plastic split rings instead. The bird was still alive in August 2012.

Metal rings may be harmful to birds if fitted above one another, as one ring may force the edges of the other to bend inwards and cause injuries. We only ringed 20 Brown Thornbills in the coastal New South Wales population with two anodized aluminium rings (2 mm inner diameter) above one another between September 2007 and April 2008. During August 2008, obvious damage to the metal colour rings was observed in all 20 individuals. We found that the edge of the rings bent inwards (Fig. 5), forming a sharp edge that constricted the leg. This problem was much more pronounced when a softer aluminium ring was mounted on top of a magnesium alloy ring. All birds ringed with anodized aluminium rings were recaptured in September 2008 and the aluminium rings were replaced by plastic rings in the upper position and a coloured aluminium ring in the lower position on the leg which did not carry the numbered alloy ring. Given that the problem which arises from mounting aluminium rings above each other was noted early enough, none of the individuals suffered from leg injuries. However, it is likely that the inwards bend edges of the rings eventually would have caused injuries.

#### (b) Metal ring size too small

We encountered this problem in Purple-crowned Fairy-wrens, which were initially ringed with size 01 rings. Out of the 90 individuals ringed with size 01 metal rings from 29 July to 11 September 2005, 4.4% of birds (four males) developed swollen ankles at the base of the metal ring within approximately a month of ringing. We removed all rings from the injured leg, which then healed rapidly. These males survived on average 2.7 years following the leg injury, from 2.4 months for a subordinate male (that could have emigrated at natal dispersal) to 5.5 years (still present at the end of the study). We were able to recapture all except 6 of the uninjured birds (that were no longer in the population), and replaced the size 01 metal ring with a size 02 metal ring.

The tarsus occupied, on average, 94% of the internal diameter of the original metal rings at its midpoint (1.88±0.01 of 2.0 mm), and 82% of the 2.3 mm internal diameter of the larger rings that we replaced them with. Tarsus width at the distal end (2.14±0.02 mm) fell between these two ring size diameters. Switching to the larger ring size dramatically reduced injury rates: over the remaining five years of the study when all birds were censused weekly, 0.04% (one male, one female) of 460 birds ringed with size 02 metal rings developed injuries on the leg with the metal ring (excluding birds ringed as nestlings that did not survive to independence at 12 weeks of age, none of which developed injuries).

### 3. Description of new colour ringing method: plastic-on-metal rings

Earlier studies found a negative impact of plastic colour rings in a number of bird species [9], [10], including thornbills [19]. Thornbills ringed with plastic colour rings developed infections at their feet leading in extreme cases to foot loss. The mechanism responsible for the infections remains unclear, but it has been speculated that not preening the leg with secretions from the uropygical gland might increase the risk of infections [10]. Thus, researchers ringing thornbills have been advised to use metal rings (numbered metal ring or plain anodized metal ring) in the basal position, adjacent to the foot [19]. However, the colour of plain anodized aluminium rings can wear off within two years, making it impossible to recognise individuals (David Green, personal communication). Based on this background, we agreed with the ABBBS to test a new technique where a plastic ring is glued onto a metal ring.

To make these plastic-on-metal rings the following materials are needed:

- Plain magnesium alloy rings of the appropriate size (same dimensions as numbered metal rings; metal rings made from other hard metals (i.e. stainless steel) can also be used). The metal rings must be slightly longer than the colour rings so that only metal touches the foot of the bird (see below). In our case we used plain alloy rings produced by Porzana (Wetland Trust, UK) with 2 mm inner diameter.
- Coloured plastic split rings with an inner diameter a little bit smaller than the outer diameter of the metal ring. We used size XCS split rings with 2.7 mm inner diameter (PVC or celluloid depending on the colour) produced by AC Hughes.
- A ringing spatula and ringing pliers
- Superglue with a drying time of 10–20 sec (we used Henkel Industry superglue; but see below regarding limitations of cyanoacrylate glues).

The rings have to be made in the field just ahead of ringing given that the plastic rings break when closing the ring once the glue is fully hardened. The plastic-on-metal rings are easily made in three steps:

1. Place the plastic ring half way onto the metal ring using a ringing spatula while keeping the openings aligned. Apply glue between the metal and plastic ring, and then push the plastic ring onto the metal ring.
2. Make sure the metal and plastic rings are level at one end. This should leave a ring of metal at the other end, which can be placed next to the foot so the plastic does not make contact with the foot (Fig. 1). Alternatively, the plastic band could be centred on the metal band (if it is sufficiently shorter), so that there is an exposed ring of metal at both the top and bottom of the band.
3. Place the open ring on the foot and close the ring with ringing pliers. If needed, apply additional glue at the opening of the colour ring to secure the 2 rings together.

Using this method, we were able to use all the colour combinations that are possible with plastic colour rings. Moreover, the plastic ring on top of the metal ring came loose only in a few cases, and the plastic ring never touched the foot. Replacing the superglue with a hard plastic adhesive (see below) might bond the plastic ring better to the metal ring since cyanoacrylate glues do not always produce long-lasting bonds (i.e. cyanoacrylate glue can deteriorate in cold climates within a year). However, we have not yet field-tested hard plastic adhesive for plastic-on-metal rings.

## Discussion

Colour ringing and other techniques used to mark birds are essential tools for learning about the ecology and population dynamics of different species. Almost all bird species tolerate plastic leg rings [7], [9], [10], and therefore this technique will remain a key method for individual recognition in field ornithology. However, in some species a proportion of individuals may react negatively to rings, requiring alternative marking methods. We discuss below in detail the different causes of ring injuries, propose alternative solutions and outline their limitations. It is important to keep in mind that all marking techniques involve some element of risk to the marked individuals, but researchers should strive to keep risks to a minimum both for animal welfare and scientific reasons. Only if we ensure that the populations under study are not affected negatively by the study methods used will the scientific findings be representative.

### 1. General advice

Field methods progress and new techniques arise [12], and it is important that researchers openly communicate both positive and negative consequences of the techniques used. Such an approach allows researchers to improve their field methodology, learn from the mistakes of others, and reduce the impact on the animals under study. However, we lack a platform to exchange this type of information, which lies outside the scope of peer-reviewed journals. Thus, we have reached an agreement with EURING (European Union for Bird Ringing) to host information regarding ringing-related damage in birds on their webpage (http://www.euring.org/research/handling_birds_responsibly.htm). A more systematic collection of ringing-related damage to birds will contribute to understanding which ecological factors influence within-species variation in sensitivity to the same method (see below), and allow appropriate ringing techniques to be chosen.

To be able to assess and compare the impact of ringing and other methods on birds, it is important to systematically record both the frequency and severity of impacts. Hence, we propose a hazard index (Table 3) to facilitate this. The index is based on a logarithmic increase of hazard points from minor inflammations (leg partly inflamed), to inflammations (whole leg inflamed), toe loss, leg damage up to the loss of a foot or a whole leg. The hazards for the study systems described here range from 0.04 (Siberian jay) to 0.11 (Purple-crowned Fairy-wrens) and 0.3 (Brown Thornbill), whereas data in the literature range from 0 (most species where ringing does not cause any injuries) to 5.4 which has been reported from the hihi *Notiomystis cincta* [30] where more than 50% of all ringed individuals had injured legs. It is important to point out that similar injuries may affect species differently depending on their ecology. Thus, this index does not give a measure of the fitness impact of ringing related injuries across species.

**Table 3:** Proposed ringing hazard categories.

Category	Hazard points	Hazard of 1 = proportion affected individuals
part of leg inflamed	1	100%
whole leg inflamed	2	50%
toe loss, foot still functional	5	20%
foot/leg deformed but still functional	10	10%
foot loss or crippled leg	20	5%
leg loss	50	2%

The hazard points proposed for the ringing hazard categories are based on the impact of the affected individuals. Given that the impact on affected individuals is likely to increase non-linearly, we suggest a logarithmic increase in hazard point between the categories.

We recommend all researchers, particularly those starting research on a new species, to inspect the feet and legs of ringed birds regularly in order to detect problems which may not appear immediately and may only affect a few birds. As our data show, in most cases only a small proportion of individuals within the population developed serious injuries. Moreover, some problems only became apparent several years after the birds were first ringed [10], making it difficult to spot problems caused by ringing. Researchers starting to colour ring a new study species should therefore monitor birds closely throughout the study, particularly species in groups where most problems with rings have been reported (thornbills, flycatchers, monarchs, small waders [8], [10], [13], [31], [32], [33]). This poses a problem for studies which only focus on one aspect of species biology, such as reproduction, for studies investigating migratory species, or short-term projects. Here a careful follow-up after colour ringing is often not feasible, which might lead to problems caused by plastic colour rings being overlooked. Small-scale trials might indicate whether plastic colour rings are suitable, although as data from our case studies highlights, sensitivity to plastic colour rings can vary between populations of the same species, and the majority of plastic colour-ringed individuals did not show any negative effects.

Depending on the problem, modifying the technique used can be a simple way to limit the harm for the birds under study (such as using coloured metal rings, using larger rings, using PMMA rings, using different glues; see below). In addition to ethical issues arising through leg problems as a consequence of colour ringing, the scientific results of studies might also be influenced. For example, in the case of Purple-crowned Fairy-wrens, the accumulation of material under the colour rings affected female breeders in particular. If all these females had died, this would have strongly influenced all results relating to reproductive output and population demography. However, even in long-term studies the long-term consequences of ringing related injuries remain largely unknown (but see [34]). If ringing negatively influences a species, the effects can be severe: a recent study on King Penguins *Aptenodytes patagonicus* demonstrated that flipper bands reduced reproductive rate by 39% and survival rate by 16% [35]. Thus, it would be valuable for other studies to investigate the long-term fitness effects of ringing on the birds under study.

It is important to keep in mind that it is costly to recapture a large number of birds to either remove or replace plastic colour rings if they are causing harm. Moreover, it is often more difficult to recapture individuals which have been caught previously. Therefore, the higher costs which arise from using an alternative technique should be balanced carefully against the costs which arise from replacing rings (including both the costs of the rings per se and the labour to recapture the birds), and the health of the birds.

### 2. Problems arising because rings are too small

A ring size that is too small can cause injuries by constricting the leg or accumulating foreign materials (e.g. shed tarsal scales [9]). Smaller birds in particular seem to develop leg injuries as a consequence of carrying leg rings (mean body weight±SE: 20.2±3.2 g, min = 7 g, max = 55 g, N = 18 species; Table 1). A possible reason for this pattern might be that in smaller birds the space between rings and the leg is relatively small, increasing the risk of material getting trapped between the rings and causing infections [9]. Our experience from Fairy-wrens shows that increasing ring size in very small species can reduce the risk of leg injuries (although in this species it was the larger (plastic) ring size that accumulated foreign material – see next section for advice on addressing that problem).

### Advice

Appropriate ring sizes are likely to vary with species depending on their behaviour and ecology. Recommended sizes range from 6% clearance [36] to around 40% clearance [9]. Our experience from Purple-crowned Fairy-wrens indicated that 6% clearance was too little for this species, with injuries developing shortly after ringing. A larger ring size with 18% clearance improved the situation. We would advise researchers working on small species to choose a larger ring with at least 15% clearance. Still, choosing a too large ring size can also be problematic, as the ring can then slip over a joint, or larger pieces of foreign material can get caught in the gap. In larger species a clearance of 15% might be too much, increasing the risk of toes getting trapped under the rings [37].

### 3. Problems due to accumulation of material under the rings

Accumulation of foreign materials (e.g. spider web in purple-crowned fairy-wrens, shed tarsal scales [9], [10], faeces [38]) under the ring are a common cause of leg injuries. Although the problem has been associated with metal bands [38], in general it seems to occur more often with plastic rings (both PVC and celluloid) (Table 1). A possible reason is that plastic may be more likely than metal to build up static and attract small pieces of tarsal scales or other organic material [9].

### Advice

Since the accumulation of material under rings is often linked to the material the ring is made of, injuries can usually be reduced or eliminated by changing the material used for ringing. Injury rates were highest on legs with two plastic rings in purple-crowned fairy-wrens, as in most other species with injuries caused by accumulation of material under the ring [9], [10], [13]. Injury rates can therefore be reduced by replacing the lower plastic ring with a metal ring (which can be coloured by anodizing), or using a split-colour metal ring [13] instead of two plastic rings (see the section below for more information on coloured metal alternatives to plastic rings; Table 4).

**Table 4:** Overview of non-plastic alternatives to colour rings.

method	pros	cons	problems for birds	recommendation
soft anodised metal rings	commercially available, cheap	colour wears off quickly	edges of rings can bend inwards if two aluminium rings are applied to one leg	only use one aluminium ring per leg; useful for sensitive species in short-term studies or where birds can be easily recaptured
hard anodised metal rings	long-lasting, split colours possible	not commercially available, expensive	edges of rings can bend inwards if two aluminium rings are applied to one leg	for long-term studies of sensitive species, use hard anodised magnesium alloy rings which are more durable than aluminium rings
pin-striping tape over metal ring	long-lasting, split colours possible	not commercially available, expensive	so far no problems observed	for long-term studies, split colouration allows many individually different colour combinations with only 2 rings
plastic-on-metal ring	long-lasting, split colours possible, cheap	requires 2–4 min longer in the field than conventional colour ringing, no long-term experience yet	heavier than a normal ring, can push plastic ring on top of tibo-tarsal joint and harm bird	cheap alternative for long-term studies, avoid using with a plastic ring on top in species which forage hanging upside down

### 4. Contact inflammations

Contact inflammations are a problem which has been reported only for plastic rings. The mechanism behind these injuries remains unclear, but some species groups are known to be sensitive to plastic colour rings (e.g. *Muscicapidae*, *Pardalotidae*, *Tyrannida*). Thus, researchers working with species in these groups are advised to consider using one of the alternatives listed in Table 4. Moreover, our results highlight that even within a species large differences can exist between populations when it comes to their sensitivity to wearing plastic rings. It remains unclear if this difference in the reaction of Brown Thornbills studied on the Australian mainland (Canberra population, coastal New South Wales population) is a consequence of ecological or genetic differences between the study populations. More data from different species would be important to understand why some species show population-specific differences in their sensitivity to plastic rings.

### Advice

Since contact inflammations are specific to plastic rings, this type of injury can be avoided by using metal rings. Hard anodized magnesium alloy rings are expensive and not commercially produced, but probably represent the best alternative to plastic rings. In contrast to anodized aluminium rings, magnesium alloy rings are much more resistant and thus the edges should not bend inwards or flatten (see below) [33]. The method suggested by Koronkiewicz et al. [13] where pin-striping tape is glued on top of a metal ring is rather laborious to produce, but no negative effects have been reported so far. The technique we present in this paper (plastic-on-metal rings) can provide a cheap alternative solution, but should not be used with a plastic ring on top if birds forage hanging upside down. We only have experience from one year in one Brown Thornbill population in Tasmania using this technique, and thus, long-term tests in other species would be valuable to assess whether plastic-on-metal rings are a useful alternative.

### 5. Toes or foot trapped under rings

This problem seems to occur mainly in species ringed with plastic wrap-around rings, but in one case it has also been reported for a species ringed with plastic split rings (hihi; [30]). Among the species listed in Table 1, those with a body weight of 35–116 g were reported to get toes or the foot trapped under plastic rings. Large birds have more powerful beaks and are thus able to pull plastic rings off. Wrap-around rings can get trapped over the foot if individuals do not manage to remove the ring completely. In addition, in larger species, toes are proportionally smaller than in smaller species, increasing the risk that toes can get caught under colour rings, as reported for the North Island Robin (Table 1) [37].

### Advice

In birds with strong bills such as jays or finches, plastic wrap-around rings should be ‘glued’, otherwise individuals risk getting a ring stuck over the foot if they try to remove it. Given that cyanoacrylate superglues do not bond PVC rings permanently, we would recommend using different plastic adhesives, such as the Hard Plastic Adhesive produced by Bison (equivalent to the UHU “Hart Kunststoff Leim”). This glue has been suggested to form a long-lasting bond (Risto Juvaste, personal communication); however, we have no personal long-term experience of this plastic adhesive. Another solution used to bond plastic rings is solvent cement, which melts PVC and therefore creates a long-lasting bond [12]. Because solvent cement is plastic-specific, it also minimizes the risk of gluing the ring to the bird and/or the ringer. Also, using wrap-around rings with more overlap (2.5 instead of 1.8–2.0 wraps like those produced commercially by AC Hughes) reduces the risk of birds being able to pull them off.

Plastic split rings only exceptionally seem to get stuck on the leg if birds manage to partially remove them. Using a battery powered soldering iron is an option to seal such rings by melting the edges together [39]. However, our field experience with another species ringed with plastic split rings (House Sparrow *Passer domesticus* [40]) showed that individuals are able to remove plastic split rings even after the split has been sealed with a soldering iron (although House Sparrows have a rather powerful bill compared to other small birds).

An alternative solution to reduce the risk of wrap-around rings or split rings being pulled off would be to use PMMA rings. These rings are more rigid than PVC rings and therefore more resistant to strong bills and are successfully used on small passerines (Pied Wagtail *Motacilla alba*, 20 g; Risto Juvaste, personal communication) [12]. If layered PMMA is used, rings can be engraved with unique codes. Using engraved rings means that only one plastic ring is needed for each bird, which is likely to minimise the impact on them [12]. This option is successfully used in a wide range of species (Risto Juvaste and Jacquie Clark, personal communications).

### 6. Problems arising through two metal rings on top of each other

If metal rings are mounted on top of each other, the edges can curl over and bend inside the ring (as in Brown Thornbills; Fig. 5), constricting the leg. This problem was pronounced if a softer aluminium ring was mounted on top of a harder magnesium alloy ring, as highlighted in Figure 5. Also, aluminium rings can gradually flatten through continuous pushing from the upper ring on the lower one. This process leads to sharp edges which can injure the legs [33]. Consequently, applying two metal rings on the same leg is not allowed in some countries [41].

### Advice

Mounting two aluminium rings on top of each other should be avoided, in particular for small species where the rings have a thin wall. We have no experience of two magnesium alloy rings mounted on top of each other, but given the harder material, this might be safer. Alternatively one metal ring coloured with pin-striping [13] could be used to give two colours on a single metal ring, an option that was effective in reducing injuries in the Purple-crowned Fairy-wren and Willow Flycatcher *Empidonax trailIii* [13].
